# Prevalence and Predictors of Intestinal Parasites among Food Handlers in Yebu Town, Southwest Ethiopia

**DOI:** 10.1371/journal.pone.0110621

**Published:** 2014-10-17

**Authors:** Tamirat Tefera, Getye Mebrie

**Affiliations:** 1 Department of Medical Laboratory Sciences and Pathology, College Of Public Health and Medical Sciences, Jimma University, Jimma, Ethiopia; 2 Yebu Health Center, Jimma zone, Ethiopia; The Australian National University, Australia

## Abstract

**Background:**

As a result of urbanization, eating and drinking from food service establishments is becoming a common practice in developing countries like Ethiopia, which increases the chances of food borne diseases. The health status and hygiene practices of food handlers are the major determinants of food contamination. In developing countries where there are poor regulatory systems for food hygiene, food handlers are often appointed without screening for possible infections associated with poor hygiene like intestinal parasites.

**Objective:**

This study aimed at determining the prevalence and predictors of intestinal parasites and assessing the hygiene practices among food handlers in Yebu Town, southwest Ethiopia.

**Methods:**

A cross-sectional study was conducted among a total of 118 food handlers in Yebu Town in January 2011. Fresh stool specimens were collected and processed using both direct wet mount and Formol ether concentration techniques.

**Results:**

The overall prevalence of intestinal parasites among the study subjects was 44.1% (52/118). *Ascaris lumbricoides* and hookworm spp were the predominant parasites identified from the stool of study participants. Age above 35 years (AOR: 4.8, 95% CI: 1.1, 21.8), no regular practice of washing hands before a meal (AOR: 7.8, 95% CI: 2.8, 24.8), and untrimmed finger nail (AOR: 14.7, 95% CI: 2.8, 75.4) were independent predictors of intestinal parasitic infection among the food handlers.

**Conclusion:**

The present study showed high prevalence of intestinal parasites among the study subjects. The study also revealed poor personal hygiene like poor practice of hand washing and poor finger nail hygiene. Therefore, much has to be done to improve the personal hygiene of the food handlers. Pre-placement and periodic screening of food handlers for parasites and prompt treatment, and health education on regular trimming or cleaning of fingernails would be the way forward for prevention of food borne diseases.

## Background

Infection with intestinal parasites remains a major public health problem of developing countries where poor environmental sanitation, poor personal hygiene and low level of education are prominent [1], [2]. Most of the intestinal parasites of medical importance are known to be transmitted by ingestion of food or water contaminated with the infective stages of these parasites [3], [4].

Globally, about one third of the total population is estimated to be infected with intestinal parasites, the majority being people living in tropical and sub-tropical parts of the world [5]. One point two billion people are infected with *Ascaris lumbricoides*, 795 million people with *Trichuris trichiura*, 740 million people with hookworm infection [6], 500 million people with *Entamoeba histolytica*, and 2.8 million people are infected with *Giardia lamblia* [7].

As in many developing countries, cases of intestinal parasitosis are highly abundant in Ethiopia. It is estimated that one third of Ethiopians are infected with *Ascaris lumbricoides*, one quarter is infected with *Trichuris trichiura* and one in eight lives with hookworm. As a result, Ethiopia has the second highest burden of ascariasis, the third highest burden of hookworm, and the fourth highest burden of trichuriasis in Sub-Saharan Africa [8].

Food borne diseases are public health problems worldwide. It was estimated that about 30% of the population living in the developed world suffers from diarrhoeal diseases, mostly caused by food borne microbial pathogens. About 2 million deaths occur annually due to food borne diseases in developing countries [9], [10].

The health status and hygiene practices of food handlers are the major determinants of food contamination. In developing countries where there is poor regulatory system for food hygiene, often these food handlers are appointed without proper screening for hygiene realted infectious diseases [11].

As a result of urbanization, eating and drinking from food service establishments is becoming a common practice in villages of developing countries like Ethiopia. Consequently, this can increase the risk of food borne diseases.

Since most intestinal helminths and protozoan parasites are transmitted through contaminated food and water [12]; food-handlers with poor personal hygiene working in food service establishments could be potential sources of infections [13].

Various studies have been conducted to assess intestinal parasitic infections among food handlers in Africa including different regions of Ethiopia. In Ethiopia, the rate of infection with intestinal parasites among food handlers ranged from 29% to 63% with different reports on the predominant species of parasites and hygiene practices [14], [15], [16]. The difference might be attributed to differences in endemicity among the towns of the country and awareness of the food handlers. Yebu Town is one of the towns found in Jimma zone, oromia regional state of Ethiopia. In this Town, the food handlers are appointed without screening for hygiene related infections like intestinal parasites. Thus, this study was designed to assess the hygiene practices, prevalence and predictors of intestinal parasites among food handlers in Yebu Town.

## Materials and Methods

### Study area and population

The study was conducted in January 2011 in Yebu Town, which is 375 km from Addis Ababa, located in south west Ethiopia. Food handlers working in different kitchens of food service establishments found in Yebu town were enrolled. A total of 118 food handlers participated in the study.

### Study design

A cross-sectional study was utilized for determination of the prevalence and predictors of intestinal parasites among food handlers in Yebu Town.

### Data collection

A pretested structured questionnaire administered by trained interviewers was used for collecting information on age, sex, educational level, access to a latrine, and hand-washing practices of each food handler. Data on shoe wearing practice and finger nail trimming of the study participants were recorded by simple observation.

### Sample size and sampling technique

Sample size was calculated using single population proportion formula assuming 95% confidence level, 5% margin of error and prevalence of 58.4% [17]. A correction formula for population less than 10,000 was applied and the final sample size was 124. The participants were selected by proportional random sampling from 20 different cafeterias and restaurants available in the Town at the time of data collection.

### Stool sample collection and processing

All of the food handlers were informed of the purpose of the study and were provided with a tight-lid plastic container after orientation on how to collect the stool specimen was given. After collection, the samples were transported to Yebu Health Center laboratory within an hour. Parasitological assessment was performed by qualified laboratory technologists using both the direct saline and iodine wet mount and Formol ether concentration techniques as described elsewhere [18]. The direct saline wet mount was employed not to miss trophozoites of *E. histolytica* and *G. lamblia*. However, in this study, no trophozoites were recorded. All of the parasites detected by direct saline wet mount were also detected by Formol ether concentration technique. Thus, the results of Formol ether concentration technique were used for analyses.

### Data analysis

Data were entered, cleaned, and analyzed using SPSS for windows version 16.0. The difference between prevalence of intestinal parasites among different categories was compared using Pearson chi-square test and Fishers exact test where appropriate. Binary logistic regression was used to identify factors associated with parasitic infection. Variables having a *p*-value of less than 0.2 in the bivariate analyses were considered for multivariate logistic regression. A *p*-value of <0.05 was used to indicate statistical significance.

### Ethical considerations

Ethical clearance was obtained from Jimma University ethical review board prior to the commencement of the study. Informed written consent was obtained from individuals who participated in the study. Individuals found to be positive for intestinal parasites were referred to the nearby health center for appropriate treatment.

## Results

A total of 118 individuals provided complete data on socio-demographic characteristics and parasitological assessments with a response rate of 95.2%. Among them, 64 (54.2%) were males and 54 (45.8%) were females. The age of the study participants ranged from 8 through 62 with a mean age of 27.5 (SD = 10.6).

The overall prevalence of intestinal parasitic infection among the food handlers was determined to be 44.1% (52/118). *A. lumbricoides* (17.8%) was the predominant parasite identified from stool of the study participants followed by hookworm spp (9.3%) (Table 1). The results of the study showed that 46.9% (30/64) of male and 40.7% (22/54) female participants were found to be infected with at least one parasite. The prevalence of infection with intestinal parasites was not significantly different among male and female food handlers (p = 0.504) see (Table 2).

**Table 1:** Frequency distribution of intestinal parasites identified from food handlers in Yebu Town.

Parasitic species	Population infected	Prevalence (%)
*A. lumbricoides*	21/118	17.8
Hookworm spp	11/118	9.3
*G. intestinalis*	7/118	5.9
*T. trichiura*	7/118	5.9
*E. histolytica/dispar/mushkoviski*	3/118	2.5
*E. vermicularis*	3/118	2.5

**Table 2:** The prevalence of intestinal parasitic infection with respect to socio-demographic characteristics of food handlers in Yebu Town.

Characteristics	N°^.^ examined	Positive	X^2^	p-value
	No (%)	No (%)		
**Gender**				
Male	64 (54.2)	30 (46.9)	0.45	0.504
Female	54 (45.8)	22 (40.7)		
**Age group**				
<20	29 (24.6)	16 (55.2)	6.04	0.049
20–35	67 (56.8)	23 (34.3)		
>35	22 (18.6)	13 (59.1)		
**Hand washing before a meal**				
Always	77 (65.3)	21 (27.3)	25.4	<0.001
Sometimes	41 (34.7)	31 (75.6)		
**Hand washing after using the toilet**				
Yes	39 (33.1)	9 (23.1)	10.4	0.001
No	79 (66.9)	43 (54.4)		
**Access to Latrine**				
Yes	117 (99.2)	51 (43.6)	1.3	0.258
No	1 (0.8)	1 (100)		
**Finger nail status**				
Trimmed	97 (82.2)	33 (34)	22.3	<0.001
Untrimmed	21 (17.8)	19 (90.5)		
**Shoe wearing habit** *				
Yes	62 (52.5)	8 (12.9)		0.211
No	56 (47.5)	3 (5.4)		
**Educational status**				
Illiterate	56 (47.5)	26 (46.4)	0.63	0.728
Primary education	50 (42.4)	20 (40)		
Secondary education	12 (10.2)	6 (50)		

*only for hookworm.

Individuals aged above 35 years of age were found to have a high percentage (59.1%) of infection as compared to other age groups. The association between age groups and intestinal parasitic infection was statistically significant (p = 0.049) (Table 3).

**Table 3:** Univariate and multivariate logistic regression analysis of predictors of intestinal parasitic infection among food handlers in Yebu Town.

Characteristics	N°^.^ examined	Positive	Crude OR (95% CI)	Adjusted OR (95%CI)
	No (%)	No (%)		
**Age group**				
<20	29 (24.6)	16 (55.2)	1.0	1.0
20–35	67 (56.8)	23 (34.3)	0.4 (0.2, 1.0)	1.1 (0.3, 3.6)
>35	22 (18.6)	13 (59.1)	1.2 (0.4, 3.6)	4.8 (1.1, 21.8)
**Hand washing before meal**				
Always	77 (65.3)	21 (27.3)	1.0	1.0
Sometimes	41 (34.7)	31 (75.6)	8.2 (3.4, 19.7)	7.8 (2.8, 24.8)
**Hand washing after toilet**				
Yes	39 (33.1)	9 (23.1)	1.0	1.0
No	79 (66.9)	43 (54.4)	3.9 (1.6, 9.4)	2.2 (0.8, 6.2)
**Finger nail status**				
Trimmed	97 (82.2)	33 (34)	1.0	1.0
Untrimmed	21 (17.8)	19 (90.5)	18.4 (4.0, 83.9)	14.7 (2.8, 75.4)

Different factors were assessed for possible association with intestinal parasitic infection among the study participants. A majority (75.6%) of individuals who had no regular practice of washing their hands before a meal were found to be infected with at least one parasite. The practice of hand washing before a meal had a statistically significant association with intestinal parasitic infection (p<0.001) (Table 2). The multivariate logistic regression model estimated that individuals who had no regular practice of washing their hands before a meal were seven times (AOR: 7.8, 95%CI: 2.8, 24.8) more likely to be infected with intestinal parasites than those who wash their hands regularly (Table 3). More than half (54.4%) of individuals who had no practice of washing hands after using the toilet were found to be infected with intestinal parasites. The practice of hand washing after using the toilet was significantly associated with parasitic infection among the study participants (p = 0.001) (Table 2). However the association was not significant after adjusting for confounders using multivariate logistic regression (Table 3).

As shown in table 1, only 10.2% (12/118) of the study participants had secondary education while 47.5% (56/118) of them had no formal education. The education level of the food handlers was not significantly associated with intestinal parasitic infection (p = 0.728).

A majority (90.5%) of individuals with untrimmed finger nails had infection with at least one parasite (Table 1). The finger nail status of the study participants had a significant association with the rate of intestinal parasitic infection (p<0.001). The odds of parasitic infection was 14 times higher (AOR: 14.7, 95%CI [2.8, 75.4]) for individuals who had not trimmed their finger nail as compared to those did (Table 3).

In this study, almost all of the study participants had access to a latrine. There was no significant association (p = 0.258) between latrine availability and infection with intestinal parasites (Table 2).

## Discussion

Several studies across the world have determined the prevalence of intestinal parasites among food handlers. The results of the present study revealed that the prevalence of intestinal parasites among the study participants was 44.1%. This is comparable with the finding of 41.1% in Bahrir Dar, Ethiopia [14], 41.2% in Enugu state, Nigeria [19], and 49.4% in Mekele, Ethiopia [16]. However it is higher than what was reported from Gondar Town, Ethiopia (29.1%) [20], Accra, Ghana (21.6%) [21], Gaza strip, Palestine (24.3%) [22], Amritsar, India (12.9%) [23], Riyadh, Saudi Arabia (12.8%) [24], and from Omdurman, Sudan (6.9%) [25]. It was much lower than the prevalence of 97% from Abeokuta, Nigeria [4], 89.6% from Minna, Nigeria [26] and 58.4% from Jimma, Ethiopia [17]. The differences might be due to differences in climate, geographical location and socio-demographic features of the populations. The prevalence among food handlers probably reflects the prevalence in the general population.

The predominant parasite identified in the present study was *A. lumbricoides* with a prevalence of 17.8%. This was consistent with the finding of a similar study conducted in Gondar Town, in which *A. lumbricoides* was the predominant parasite reported with a prevalence of 18.1% [20].

In the present study, the practice of hand washing after using the toilet among the food handlers was very poor (33.1%), and much poorer than is conducted in Bahir Dar Town, Ethiopia (90.6%) [14] and in Maharashtra, India (49.38%) [27].

The multivariate logistic regression analysis showed that hand washing before a meal, finger nail status and age group were independent predictors of intestinal parasitism among the food handlers found in Yebu Town. Other studies have also shown hand washing practice to be a determinant for intestinal parasitic infection among food handlers [14], [16]. Examination of finger nail contents of food handlers for ova or parasites is one way of indicating the possible contamination of food [26], [28]. Nevertheless the present study did not attempt to assess the parasite carriage of the finger nail contents.

## Conclusion

This study revealed a high prevalence of intestinal parasites among food handlers in Yebu Town. Since most of the intestinal parasites are transmitted by the feco-oral route, food handlers could be an important source of infection to the general population. The study also identified finger nail status and hand washing before a meal as determinants of intestinal parasitic infection. Therefore much has to be done to improve the personal hygiene of the food handlers. Pre-placement and periodic screening of food handlers for parasites, periodic deworming, health education on regular trimming or cleaning of fingernails and training of food handlers in basic principles of hygienic food handling would be the way forward for prevention of food borne diseases in Ethiopia.
